# Rotigotine transdermal system and evaluation of pain in patients with Parkinson’s disease: a *post hoc* analysis of the RECOVER study

**DOI:** 10.1186/1471-2377-14-42

**Published:** 2014-03-06

**Authors:** Jan Kassubek, Kallol Ray Chaudhuri, Theresa Zesiewicz, Erwin Surmann, Babak Boroojerdi, Kimberly Moran, Liesbet Ghys, Claudia Trenkwalder

**Affiliations:** 1 Department of Neurology, University of Ulm, Oberer Eselsberg 45, 89081 Ulm, Germany; 2 National Parkinson Foundation Centre of Excellence, King’s College Hospital, London, UK; 3 MRC Centre for Neurodegeneration Research, King’s College, London, UK; 4 University of South Florida, Tampa, FL, USA; 5 UCB Pharma, Monheim am Rhein, Germany; 6 UCB Pharma, Raleigh, NC, USA; 7 UCB Pharma, Smyrna, GA, USA; 8 UCB Pharma, Brussels, Belgium; 9 University of Göttingen, Department of Neurosurgery and Paracelsus-Elena Klinik, Center of Parkinsonism and Movement Disorders, Kassel, Germany

**Keywords:** Parkinson's disease, Pain, Rotigotine, Dopamine receptor agonist

## Abstract

**Background:**

Pain is a troublesome non-motor symptom of Parkinson’s disease (PD). The RECOVER (Randomized Evaluation of the 24-hour Coverage: Efficacy of Rotigotine; Clintrials.gov: NCT00474058) study demonstrated significant improvements in early-morning motor function (UPDRS III) and sleep disturbances (PDSS-2) with rotigotine transdermal system. Improvements were also reported on a Likert pain scale (measuring any type of pain). This *post hoc* analysis of RECOVER further evaluates the effect of rotigotine on pain, and whether improvements in pain may be attributable to benefits in motor function or sleep disturbance.

**Methods:**

PD patients with unsatisfactory early-morning motor impairment were randomized to optimal-dose (up to 16 mg/24 h) rotigotine or placebo, maintained for 4 weeks. Pain was assessed in the early-morning using an 11-point Likert pain scale (rated average severity of pain (of any type) over the preceding 12 hours from 0 [no pain] to 10 [worst pain ever experienced]). *Post hoc* analyses for patients reporting ‘any’ pain (pain score ≥1) at baseline, and subgroups reporting ‘mild’ (score 1–3), and ‘moderate-to-severe’ pain (score ≥4) were performed. Likert pain scale change from baseline in rotigotine-treated patients was further analyzed based on a UPDRS III/PDSS-2 responder analysis (a responder defined as showing a ≥30% reduction in early morning UPDRS III total score or PDSS-2 total score). As *post hoc* analyses, all p values presented are exploratory.

**Results:**

Of 267 patients with Likert pain data (178 rotigotine, 89 placebo), 187 (70%) reported ‘any’ pain; of these 87 (33%) reported ‘mild’, and 100 (37%) ‘moderate-to-severe’ pain. Change from baseline pain scores decreased with rotigotine compared with placebo in patients with ‘any’ pain (-0.88 [95% CI: -1.56, -0.19], p = 0.013), and in the subgroup with ‘moderate-to-severe’ pain (-1.38 [-2.44, -0.31], p = 0.012). UPDRS III or PDSS-2 responders showed greater improvement in pain than non-responders.

**Conclusions:**

The results from this *post hoc* analysis of the RECOVER study suggest that pain was improved in patients with PD treated with rotigotine; this may be partly attributable to benefits in motor function and sleep disturbances. Prospective studies are warranted to investigate this potential benefit and the clinical relevance of these findings.

## Background

Pain is a common and challenging non-motor symptom of Parkinson’s disease (PD), occurring in 40% to 85% of patients with PD [1], with a higher prevalence and intensity than in age-matched non-PD controls [1-5]. Pain is rated by patients as one of the most troublesome symptoms in both early and advanced stages of PD [6], and is associated with reduced health-related quality of life [6-10]. The complexity of PD-associated pain is exemplified by the many different types and distributions of pain, and the poor understanding of the mechanisms of the pain syndrome. The origin of pain in PD may be directly attributable to the patient’s motor symptoms such as rigidity, dystonia, akinesia, or postural abnormalities. Musculoskeletal pain is the most commonly reported type of pain (by up to 70% of patients [1,2,11]) and may be related to the presence of rigidity and akinesia, as well as comorbid rheumatologic and orthopedic diseases, which can result from pathologic postures such as camptocormia [12]. Pain associated with dystonic symptoms, including spasms, has been reported by up to 40% of PD patients [1,2,11] and is often associated with levodopa wearing off as the disease progresses, particularly in the early morning [13]. In addition to pain being secondary to motor symptoms, data also suggest that altered central pain processing in PD may lead to a decreased pain threshold and abnormal pain-evoked response, resulting in a predisposition to develop pain [11,13,14].

Rotigotine is a dopamine receptor agonist with activity across D1 through D5 receptors as well as select adrenergic and serotonergic sites [15]; continuous transdermal delivery of rotigotine maintains stable plasma levels over 24 hours with a single daily application [16]. The RECOVER (Randomized Evaluation of the 24-hour Coverage: Efficacy of Rotigotine; Clintrials.gov: NCT00474058) study demonstrated that 4 weeks maintenance with rotigotine transdermal system significantly improved the co-primary outcome measures, i.e., early morning motor function and nocturnal sleep disturbances, in PD patients with unsatisfactory control of early morning motor symptoms [17]. In addition, improvements were reported for other non-motor outcome measures, including a standard Likert pain scale (measuring any type of pain), the Non-Motor Symptoms Scale (NMSS) total and domain scores, the short form 8-item Parkinson’s Disease Questionnaire (PDQ-8) total score, and the Nocturnal Akinesia, Dystonia and Cramps (NADCS) total score [17].

The current *post hoc* analysis of the RECOVER study further investigates the effect of rotigotine transdermal system on pain among patients with PD who reported pain at baseline as rated by a Likert pain scale. *Post hoc* analyses were also performed to investigate the possibility that the observed improvements in pain with rotigotine may at least in part, be directly attributable to the improvement in motor impairment and sleep disturbance.

## Methods

### Patient eligibility

Enrolled patients were men and women aged 18 years or older, with PD (Hoehn and Yahr Stages I–IV), and unsatisfactory control of early morning motor symptoms as determined by the investigator. Immediate-release levodopa was permitted provided the dose was stable for at least 28 days prior to baseline. The full RECOVER (Clintrials.gov: NCT00474058) study design including complete inclusion/exclusion criteria has been published [17]. The study was conducted in accordance with Good Clinical Practice and the Declaration of Helsinki. The study protocol and amendments were approved by the national, regional, or Independent Ethics Committees or Institutional Review Boards of all 49 participating centres (in 12 countries) (Additional file 1). All patients provided written, informed consent before study participation.

### Procedures

Patients were randomized 2:1 to receive transdermal patches containing rotigotine or placebo; treatment was titrated to optimal dose (defined as the dose at which both the investigator and patient felt that early morning motor impairment was adequately controlled) over 1 to 8 weeks, starting at 2 mg/24 h and increasing to a maximum of 16 mg/24 h. This dose was maintained for 4 weeks (maintenance phase), during which dose adjustments (and alteration of levodopa dose) were not permitted. Visits were scheduled at screening and baseline; every 2 weeks during dose titration; start and end of maintenance; and 30 days after treatment ended. Patients were hospitalized for two nights at baseline and at the end of maintenance. Patients who withdrew prematurely were asked to return for a withdrawal visit. All efficacy outcomes were assessed in the early morning; they were assessed at baseline before new patch application (after the first or second night of hospitalization), and at the end of maintenance, or in the event that a patient was prematurely withdrawn from the trial.

### Assessment of pain

Patient rating of pain was recorded in the early morning, after the second night of hospitalization, using a standard 11-point Likert pain scale [18]. The scale rated the patient’s average severity of pain (of any type) over the preceding 12 hours from 0 (no pain) to 10 (worst pain ever experienced). In this *post hoc* analysis, Likert pain scale score change from baseline to end of treatment was assessed in patients with ‘any pain’ at baseline, which was defined as those who recorded a baseline Likert pain scale score ≥1. Two further subgroups were defined from this group: patients reporting ‘mild pain’ (Likert pain scale score 1–3), and those reporting ‘moderate-to-severe pain’ (Likert pain scale score ≥4) at baseline.

Six items were identified within the other assessment scales used in RECOVER, that also provide an evaluation of pain: ‘nocturnal cramps’ (NADCS) [19]; ‘painful muscle cramps or spasms due to PD’ (PDQ-8) [20]; nocturnal ‘pain in arms or legs’, ‘muscle cramps in arms or legs’, and ‘painful posturing in the morning’ (Parkinson’s Disease Sleep Scale [PDSS-2]) [21]; and ‘suffer from pain not explained by other known conditions’ (NMSS) [22]. These were assessed in the early morning. Baseline scores of these individual items were assessed in patients stratified by the severity of overall pain at baseline for the subgroups with ‘no pain’, ‘mild pain’, and ‘moderate-to-severe pain’, as rated by the Likert pain scale.

The co-primary efficacy measures in RECOVER were change from baseline in early morning motor function assessed using the Unified Parkinson’s Disease Rating Scale (UPDRS) Part III (Motor Examination) measured in the early morning, and nocturnal sleep disturbances assessed using the modified PDSS-2 total scores. To investigate the possibility that the observed improvements in pain with rotigotine may be directly attributable to the improvement in motor function and sleep disturbance, the Likert pain scale change from baseline in rotigotine-treated patients was further analyzed based on a UPDRS III/PDSS-2 responder analysis. In RECOVER, a patient was defined as a responder if showing a ≥30% reduction [23,24] in early morning UPDRS III total score or PDSS-2 total score from baseline to end of maintenance. Associations of change in early morning motor function and nocturnal sleep disturbances with change in pain were also assessed using Pearson correlation coefficients for change from baseline to end of maintenance in rotigotine-treated patients with pain at baseline (as rated by the Likert pain scale).

### Statistical analyses

Likert pain scale was assessed as change from baseline to end of treatment, where end of treatment was the combined results of observations from end of maintenance and early withdrawal, using data as observed. Likert pain scale analyses were performed on the full analysis set (FAS). The FAS included all randomized patients who received at least one dose of study drug and had a baseline and at least one post-baseline measurement for both co-primary efficacy variables. UPDRS Part III and PDSS-2 were performed on the FAS and were assessed as change from baseline to end of maintenance, with last observation carried forward. Differences in demographics/baseline characteristics between patients with pain and patients with no pain at baseline (as assessed using the Likert pain scale) were estimated using t-test for continuous data, and the Chi-square test for categorical data. Treatment differences for Likert pain score change from baseline to end of treatment were estimated using analysis of covariance (ANCOVA), with treatment and region as factors and baseline Likert pain value as the covariate. UPDRS and PDSS-2 30% responder status differences for Likert pain score change from baseline to end of treatment were estimated using ANCOVA, with responder status and region as factors and baseline Likert pain value as the covariate. As *post hoc* analyses, all p values presented are exploratory and do not infer statistical significance. No adjustments were made for multiplicity due to closed test procedure for primary efficacy variables.

## Results

### Patient disposition and baseline characteristics

Of 287 patients randomized (97 placebo, 190 rotigotine), 246 completed the study; 267 patients (89 placebo, 178 rotigotine) were included in the FAS, all of whom had baseline and follow-up Likert pain scale scores. At baseline, 187 (70%) patients with a Likert pain scale score ≥1 were considered to have ‘any pain’ at baseline; 100 (37%) patients with a Likert pain scale score ≥4 were considered to have ‘moderate-to-severe’ pain (Table 1). Of these patients, 21 reported ‘severe’ pain (defined as Likert pain scale score ≥7); this subgroup with ‘severe’ pain was not investigated further due to the small sample size, but was included in the overall analyses. Patients with ‘moderate-to-severe’ pain at baseline had higher baseline scores on UPDRS III (p = 0.013) and PDSS-2 (p < 0.0001) than patients without pain (Table 1).

**Table 1:** Demographic/baseline characteristics: by presence/absence of pain at baseline (as rated by Likert pain scale)

	**‘No’ pain (n = 80) (pain score 0)**	**‘Any’ pain (n = 187)**	
		**(pain score ≥1)**	
		**‘Mild’ pain (n = 87)**	**‘Moderate-to-severe’ pain (n = 100)**
		**(pain score 1–3)**	**(pain score ≥4)**
Age, years	66.4 ± 9.3 (37–86)	63.8 ± 9.8 (37–85); (p = 0.048)	
		63.1 ± 10.4 (37–85); (p = 0.032)	64.5 ± 9.3 (40–83); (p = 0.175)
Female	23 (29)	69 (37); (p = 0.199)	
		23 (26); (p = 0.738)	46 (46); (p = 0.018)
Time since diagnosis, years	4.7 ± 4.4 (0.0–22.9)	4.9 ± 4.4 (0.0–25.6); (p = 0.820)	
		4.5 ± 3.8 (0.0–17.2); (p = 0.765)	5.1 ± 4.9 (0.0–25.6); (p = 0.555)
Taking levodopa (advanced PD)	62 (78)	156 (83); (p = 0.252)	
		70 (80); (p = 0.639)	86 (86); (p = 0.138)
UPDRS III total score	28.3 ± 11.1	31.3 ± 13.5; (p = 0.075)	
		29.2 ± 11.9; (p = 0.621)	33.2 ± 14.5; (p = 0.013)
PDSS-2 total score	16.0 ± 7.8	21.2 ± 9.8; (p <0.0001)	
		18.6 ± 9.2; (p = 0.048)	23.5 ± 9.8; (p <0.0001)

Data are mean ± SD (range) or number of patients (%). Data are presented for the FAS.

P values (t-test for continuous data, Chi-square test for categorical data; exploratory analyses) are reported for patients with ‘Any’ pain, ‘Mild’ pain, and ‘Moderate-to-severe’ pain vs patients with ‘No’ pain at baseline (assessed by the Likert pain scale).

FAS: full analysis set; PD: Parkinson’s disease; PDSS-2: Parkinson’s Disease Sleep Scale; SD: standard deviation; UPDRS III: Unified Parkinson’s Disease Rating Scale.

The mean ± SD rotigotine dose during the RECOVER study maintenance period for the different Likert pain scale subgroups were: 10.3 ± 4.8 mg/24 h (n = 46) for patients reporting ‘no pain’ at baseline (pain score = 0), 11.5 ± 4.3 mg/24 h (n = 132) for patients reporting ‘any’ pain’, 12.0 ± 3.9 mg/24 h (n = 62) for patients reporting ‘mild’ pain, and 11.1 ± 4.6 mg/24 h (n = 70) for patients reporting ‘moderate-to-severe’ pain.

At baseline, individual item scores from other scales used in RECOVER assessing pain, i.e., NADCS, PDQ-8, PDSS-2, and NMSS, were higher (i.e., specific pain more frequent/more severe) in patients with greater severity of pain as assessed by the Likert pain scale (Table 2). Change from baseline data for all items of the PDQ-8 and PDSS-2 showed an improvement with rotigotine versus placebo in those items assessing pain and are reported elsewhere [25].

**Table 2:** Baseline scores of items assessing pain in RECOVER by severity of overall pain (Likert pain scale)

	**‘Nocturnal cramps’ (from NADCS)**	**‘Painful muscle cramps or spasms due to PD’ (PDQ-8 item 7)**	**‘Pain in arms or legs’ (PDSS-2 item 10)**	**‘Muscle cramps in arms or legs’ (PDSS-2 item 11)**	**‘Painful posturing in the morning’ (PDSS-2 item 12)**	**‘Suffer from pain not explained by other known conditions’ (NMSS item 29)**
**‘No’ pain (n = 80)** (pain score 0)	0.4 ± 0.8	0.8 ± 0.9	0.5 ± 0.9	0.6 ± 1.0	0.5 ± 0.9	0.6 ± 1.8
**‘Mild’ pain (n = 87)** (pain score 1–3)	0.7 ± 0.9 (p = 0.020)	1.3 ± 1.0 (p < 0.001)	1.1 ± 1.1 (p <0.001)	1.0 ± 1.1 (p = 0.007)	0.7 ± 1.0 (p = 0.139)	1.1 ± 2.1 (p = 0.062)
**‘Moderate-to-severe’ pain (n = 100)** (pain score ≥4)	1.1 ± 1.1 (p <0.0001)	1.7 ± 1.0 (p <0.0001)	1.9 ± 1.2 (p <0.0001)	1.5 ± 1.2 (p <0.0001)	1.4 ± 1.2 (p <0.0001)	2.1 ± 3.4 (p <0.001)

Data are mean ± SD. Data are presented for the FAS.

P values (t-test exploratory analyses) are reported for patients with ‘Mild’ pain and patients with ‘Moderate-to-severe’ pain vs patients with ‘No’ pain at baseline (assessed by the Likert pain scale).

FAS: full analysis set; NADSC: Nocturnal Akinesia, Dystonia and Cramps total score; NMSS: Non-Motor Symptoms Scale; PD: Parkinson’s disease; PDQ-8: 8-item Parkinson’s Disease Questionnaire; PDSS-2: Parkinson’s Disease Sleep Scale; RECOVER: Randomized Evaluation of the 24-hour Coverage: Efficacy of Rotigotine; SD: standard deviation.

### Efficacy outcomes

**Figure 1:**
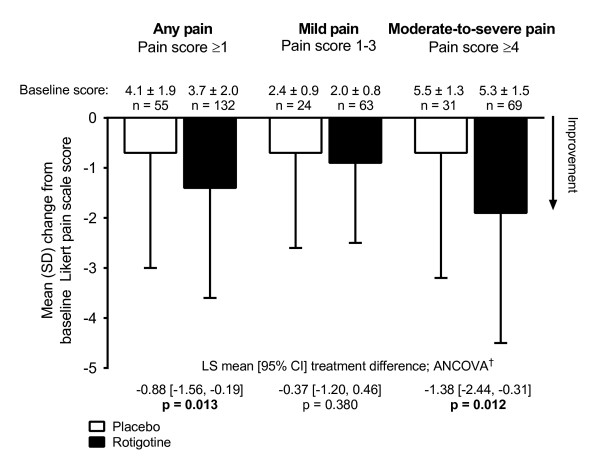
**Likert pain scale change from baseline in patients with pain at baseline.**^†^ANCOVA model with treatment and region as factors and baseline pain score as the covariate. ANCOVA: analysis of covariance; CI: confidence interval; LS: least square; SD: standard deviation.

In patients with ‘any’ pain at baseline as rated by the Likert pain scale, change in Likert pain score from baseline to end of treatment decreased with rotigotine treatment compared with placebo, with a least squares (LS) mean [95% CI] treatment difference of -0.88 [-1.56, -0.19], p = 0.013. This was also the case in the subgroup of patients with ‘moderate-to-severe’ pain (-1.38 [CI: -2.44, -0.31], p = 0.012), but was not observed in patients with ‘mild’ pain (Figure 1).

**Figure 2:**
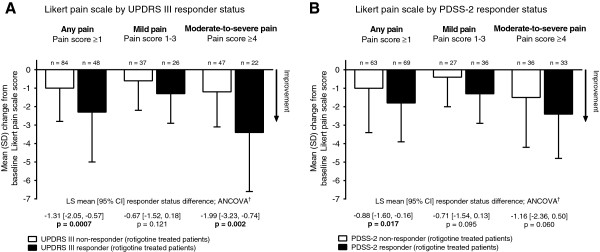
**Likert pain scale change from baseline in rotigotine-treated patients by A. UPDRS III responder status, and B. PDSS-2 responder status.**^†^ANCOVA model with UPDRS III/PDSS-2 responder status and region as factors and baseline pain score as the covariate. ANCOVA: analysis of covariance; CI: confidence interval; LS: least square; PDSS-2: Parkinson’s Disease Sleep Scale; SD: standard deviation; UPDRS III: Unified Parkinson’s Disease Rating Scale.

Rotigotine-treated patients defined as UPDRS Part III 30% responders, showed a greater improvement on the Likert pain scale than the non-responders. This was the case for the patient group reporting ‘any pain’ at baseline (LS mean [95% CI] responder status difference -1.31 [-2.05, -0.57], p = 0.0007) and for the subgroup reporting ‘moderate-to-severe’ pain at baseline (-1.99 [-3.23, -0.74], p = 0.002) (Figure 2A). Similarly, rotigotine-treated patients classified as PDSS-2 30% responders showed a greater improvement on the pain scale than the non-responders (Figure 2B). Pearson correlation coefficients (*r*) between change from baseline to end of maintenance in UPDRS III and PDSS-2 with Likert pain scale score change from baseline (rotigotine-treated patients) are presented in Additional file 2: Table S1; there was a small association between change in pain and changes in motor function (*r* = 0.40 [patients with ‘mild’ pain], and *r* = 0.31 [patients with ‘moderate-to-severe’ pain]) and nocturnal sleep disturbances (*r* = 0.30 [patients with ‘mild’ pain], and *r* = 0.16 [patients with ‘moderate-to-severe’ pain]). Results were comparable for Spearman rank-order correlation coefficients (data not shown).

## Discussion

Our results show that more than two-thirds (70%) of patients who participated in the RECOVER study reported experiencing pain at baseline, in the 12 hours preceding the early-morning assessments as rated by the Likert pain scale (score ≥1). Over a third (37%) reported ‘moderate-to-severe’ pain (score ≥4), supporting prior observations documenting pain as a common non-motor symptom of PD [1-5]. The efficacy results from this *post hoc* analysis suggest that 4 weeks of treatment with optimal-dose rotigotine, up to 16 mg/24 h, improved pain in patients reporting ‘any’ pain at baseline, and in the subgroup reporting ‘moderate-to-severe’ pain.

The Likert pain scale indicates both presence and severity of pain without distinguishing different types of pain or causality. However, of note, patients who reported pain at baseline appeared to have more advanced motor symptoms of PD based on higher UPDRS Part III score (p = 0.013 in patients with ‘moderate-to-severe’ pain vs patients with ‘no’ pain), and greater nocturnal sleep disturbance, as assessed by PDSS-2 (p <0.05 in patients with ‘any’ pain, ‘mild’ pain and ‘moderate-to-severe’ pain vs patients with ‘no’ pain). In addition, the proportion of patients receiving levodopa was numerically larger and mean disease duration was longer than in patients who did not report ‘any’ pain at baseline (p >0.05). This would suggest that the symptoms of pain observed at baseline in these patients may, at least in part, be related to the severity of symptoms of PD. There did not appear to be any difference in mean rotigotine maintenance dose between the different pain subgroups. The most marked rotigotine treatment effect, i.e., improvement in pain, was seen in patients with the most pronounced pain, i.e., the ‘moderate-to-severe’ pain subgroup, while no relevant improvement compared with placebo was observed in the subgroup of patients with ‘mild’ pain. However, the number of patients in this subgroup with ‘mild’ pain may not have been sufficient to detect an effect for only mild symptoms. In addition, by definition, patients in this subgroup had lower baseline Likert pain scores (i.e., score 1–3), and there was therefore less scope to detect a treatment effect.

Patients who responded to treatment with rotigotine with at least a 30% improvement in their early morning motor function (UPDRS Part III) or nocturnal sleep disturbances (PDSS-2) showed a greater improvement in overall pain compared with the UPDRS III/ PDSS-2 non-responders, supporting the concept that the pain reported was to some extent related to their symptoms of PD. In addition, baseline scores of the individual items of the other scales used in RECOVER that assessed pain (from the NADCS, PDQ-8, PDSS-2, and NMSS) were higher in patients with pain as assessed by the Likert pain scale than in patients with no pain (p <0.001 in patients with ‘moderate-to-severe’ pain vs patients with ‘no’ pain for all individual items assessing pain). This again suggests that the pain measured by the Likert pain scale was likely to capture types of pain assessed with these items, e.g., pain in limbs, muscle cramps due to PD, and painful posturing. A previous *post hoc* analysis of the RECOVER study, evaluating individual items of the PDSS-2 and PDQ-8, showed that the prevalence of four items recording the presence of pain ranged from 27% (painful posturing in the morning) to 38% (painful muscle cramps or spasms), and suggested that rotigotine had a beneficial effect on ‘painful muscle cramps or spasms due to PD’, frequency of nocturnal ‘pain in arms or legs’, nocturnal ‘muscle cramps in arms or legs’, and ‘painful posturing in the morning’ [25]. These symptoms can be signs of the ‘off’ state of PD, which may have predominated at that time, and possibly be associated with levodopa wearing off. All patients in the RECOVER study had inadequate control of early morning motor function. It is therefore possible that patients who were taking levodopa may have been under dosed, and this may also be a factor contributing to the pain reported in these patients. Taken together, these data suggest that the improvement in pain with rotigotine may, at least in part, be secondary to improvements in motor symptoms, including dystonic symptoms at night or in the early morning.

Pain in patients with PD has previously been described to fluctuate with motor fluctuations, with more frequent and severe pain in ‘off’ compared with ‘on’ states [26]. However, as the correlation analyses in the current study showed a small association between change in pain and changes in motor function (*r* = 0.31–0.40) and nocturnal sleep disturbances (*r* = 0.16–0.30), the pain reported by the patients may also include pain unrelated to motor function or sleep disturbance. Pain in PD should therefore be considered as a non-motor symptom in PD *per se*, and may also be linked to alterations in pain sensation [11,13,14,27,28]. Although the extent of dopaminergic neuron involvement in pain perception is not established, studies have suggested that dopaminergic stimulation of striatal dopamine D2/D3 receptors may improve pain [29].

In the RECOVER study population, improvements were also observed in favor of rotigotine in scales/items assessing the neuropsychiatric symptoms of depression, apathy and anhedonia (secondary or *post hoc* exploratory analyses) [17,30]. It is possible that elevated mood may have contributed to improvements in subjective pain and, conversely, improvements in pain may have contributed to improvements in mood. However, it is not possible to characterize the interaction between the observed improvements in pain and neuropsychiatric symptoms from this *post hoc* analysis.

There are several limitations of this *post hoc* analysis to consider. First, these analyses are exploratory in nature therefore the statistical significance or clinical relevance of the observed improvements in pain, following treatment with rotigotine cannot be determined from these results. Second, because patients were not selected for the RECOVER study based on symptoms of pain, *post hoc* selection using subjective Likert pain scales scores may have biased the results. Third, the limitation of the Likert pain scale to assess general pain, i.e., its inability to record information on the types and causes of pain, and also the retrospective observation limited to the preceding 12 hours. It is also important to note that the clinical relevance of the arbitrary categorization of patients as reporting ‘mild’ and ‘moderate-to-severe’ pain on the Likert pain scale has not been defined. However, as there is currently no validated pain scale for patients with PD, we relied on a standard Likert pain scale, accepting that this scale is not specific to PD-related or associated pain.

## Conclusions

Despite the limitations of this *post hoc* analysis, these data support existing information that pain is a prevalent non-motor symptom associated with PD. This study has begun to characterize some of the types (e.g., cramps, spasms, painful posturing) and distribution of pain (e.g., pain in limbs) that may be improved with rotigotine; our results also suggest that the improvement in pain was to some extent attributable to the benefits in motor function and nocturnal sleep disturbances, such as dystonic symptoms at night or in the early morning. Prospective studies are warranted to investigate the potential improvements in pain with rotigotine transdermal system, and to determine the clinical relevance of these findings.

## Competing interests

JK has received personal compensation for activities with UCB Pharma, GlaxoSmithKline, Teva, Medtronic, and Boehringer Ingelheim Pharmaceuticals, Inc. as a consultant and honoraria for academic lectures from Merz and Bayer. KRC serves as the European Editor of *Basal Ganglia*, as an editorial board member of *Parkinsonism and Related Disorders*, and the *Journal of Parkinson’s Disease*, and as the Liaison and PR Committee Chairman of the *Movement Disorder Society*; has received honoraria for academic lectures at sponsored symposiums from UCB Pharma, Britannia, GlaxoSmithKline, Abbott, Teva, Medtronic, and Boehringer Ingelheim Pharmaceuticals, Inc.; and has received educational grants for research from UCB Pharma, Abbott, Boehringer Ingelheim Pharmaceuticals, Inc., and Britannia. TAZ has received compensation from UCB Pharma, GlaxoSmithKline, Teva, Edison Pharmaceuticals, General Electric, and the Friedreich’s Ataxia Research Alliance (FARA). ES is an employee of UCB Pharma, Monheim am Rhein, Germany. BB is an employee of UCB Pharma, Raleigh, NC, USA and receives stock options from this employment. KM is an employee of UCB Pharma, Smyrna, GA, USA and receives stock options from this employment. LG was an employee of UCB Pharma, Brussels, Belgium, during the development of the manuscript. CT is a consultant for and/or has received honoraria/research support from Vifor Pharma, UCB Pharma, Mundipharma, Britannia, Novartis, Boehringer Ingelheim Pharmaceuticals, Inc., GlaxoSmithKline, Teva Neuroscience and Desitin.

## Authors’ contributions

JK, KRC, TZ, BB, and CT participated in the analysis and interpretation of data, and were involved in revising the manuscript critically for important intellectual content. ES participated in the design of the current *post hoc* analyses, performed the statistical analysis, and was involved in revising the manuscript critically for important intellectual content. KM and LG participated in the design of the current *post hoc* analyses, participated in the analysis and interpretation of data, and were involved in revising the manuscript critically for important intellectual content. All authors read and approved the final manuscript.

## Pre-publication history

The pre-publication history for this paper can be accessed here:

http://www.biomedcentral.com/1471-2377/14/42/prepub

## Supplementary Material

Additional file 1 Independent Ethics Committees or Institutional Review Boards of participating centres in the RECOVER study. Click here for file

Additional file 2: Table S1 Pearson correlation coefficients (*r*) for change from baseline in Likert pain scale score with UPDRS III and PDSS-2 change from baseline in rotigotine-treated patients. Click here for file

## References

[B1] Broen MP Braaksma MM Patijn J Weber WE Prevalence of pain in Parkinson’s disease: a systematic review using the modified QUADAS tool Mov Disord 2012 27 480 484 22231908 10.1002/mds.24054

[B2] Beiske AG Loge JH Rønningen A Svensson E Pain in Parkinson’s disease: Prevalence and characteristics Pain 2009 141 173 177 19100686 10.1016/j.pain.2008.12.004

[B3] Broetz D Eichner M Gasser T Weller M Steinbach JP Radicular and nonradicular back pain in Parkinson’s disease: a controlled study Mov Disord 2007 22 853 856 17357131 10.1002/mds.21439

[B4] Defazio G Berardelli A Fabbrini G Martino D Fincati E Fiaschi A Moretto G Abbruzzese G Marchese R Bonuccelli U Del Dotto P Barone P De Vivo E Albanese A Antonini A Canesi M Lopiano L Zibetti M Nappi G Martignoni E Lamberti P Tinazzi M Pain as a nonmotor symptom of Parkinson disease: evidence from a case-control study Arch Neurol 2008 65 1191 1194 18779422 10.1001/archneurol.2008.2

[B5] Nègre-Pagès L Regragui W Bouhassira D Grandjean H Rascol O Chronic pain in Parkinson’s disease: the cross-sectional French DoPaMiP survey Mov Disord 2008 23 1361 1369 18546344 10.1002/mds.22142

[B6] Politis M Wu K Molloy S G. Bain P Chaudhuri KR Piccini P Parkinson’s disease symptoms: the patient’s perspective Mov Disord 2010 25 1646 1651 20629164 10.1002/mds.23135

[B7] Gallagher DA Lees AJ Schrag A What are the most important nonmotor symptoms in patients with Parkinson’s disease and are we missing them? Mov Disord 2010 25 2493 2500 20922807 10.1002/mds.23394

[B8] Quittenbaum BH Grahn B Quality of life and pain in Parkinson’s disease: a controlled cross-sectional study Parkinsonism Relat Disord 2004 10 129 136 15036166 10.1016/j.parkreldis.2003.12.001

[B9] Martinez-Martin P The importance of non-motor disturbances to quality of life in Parkinson’s disease J Neurol Sci 2011 310 12 16 21621226 10.1016/j.jns.2011.05.006

[B10] Roh JH Kim BJ Jang JH Seo WK Lee SH Kim JH Oh K Park KW Lee DH Koh SB The relationship of pain and health-related quality of life in Korean patients with Parkinson’s disease Acta Neurol Scand 2009 119 397 403 18976321 10.1111/j.1600-0404.2008.01114.x

[B11] Ha AD Jankovic J Pain in Parkinson’s disease Mov Disord 2012 27 485 491 21953990 10.1002/mds.23959

[B12] Doherty KM van de Warrenburg BP Peralta MC Silveira-Moriyama L Azulay JP Gershanik OS Bloem BR Postural deformities in Parkinson’s disease Lancet Neurol 2011 10 538 549 21514890 10.1016/S1474-4422(11)70067-9

[B13] Wasner G Deuschl G Pains in Parkinson disease many syndromes under one umbrella Nat Rev Neurol 2012 8 284 294 22508236 10.1038/nrneurol.2012.54

[B14] Ford B Pain in Parkinson’s disease Mov Disord 2010 25 S98 S103 20187254 10.1002/mds.22716

[B15] Stockwell KA Scheller D Rose S Jackson MJ Tayarani-Binazir K Iravani MM Smith LA Olanow CW Jenner P Continuous administration of rotigotine to MPTP-treated common marmosets enhances anti-parkinsonian activity and reduces dyskinesia induction Exp Neurol 2009 219 533 542 19619533 10.1016/j.expneurol.2009.07.011

[B16] Elshoff JP Braun M Andreas JO Middle M Cawello W Steady-state plasma concentration profile of transdermal rotigotine: an integrated analysis of three, open-label, randomized, phase I multiple dose studies Clin Ther 2012 34 966 978 22401642 10.1016/j.clinthera.2012.02.008

[B17] Trenkwalder C Kies B Rudzinska M Fine J Nikl J Honczarenko K Dioszeghy P Hill D Anderson T Myllyla V Kassubek J Steiger M Zucconi M Tolosa E Poewe W Surmann E Whitesides J Boroojerdi B Chaudhuri KR Recover Study Group Rotigotine effects on early morning motor function and sleep in Parkinson’s disease: a double-blind, randomized, placebo-controlled study (RECOVER) Mov Disord 2011 26 90 99 21322021 10.1002/mds.23441 PMC3072524

[B18] Plan EL Elshoff JP Stockis A Sargentini-Maier ML Karlsson MO Likert pain score modeling: a Markov integer model and an autoregressive continuous model Clin Pharmacol Ther 2012 91 820 828 22433987 10.1038/clpt.2011.301

[B19] Stocchi F Barbato L Nordera G Berardelli A Ruggieri S Sleep disorders in Parkinson’s disease J Neurol 1998 245 S15 S18 9617717 10.1007/pl00007731

[B20] Peto V Jenkinson C Fitzpatrick R PDQ-39: a review of the development, validation and application of a Parkinson’s disease quality of life questionnaire and its associated measures J Neurol Sci 1998 245 S10 S14 10.1007/pl00007730 9617716

[B21] Trenkwalder C Kohnen R Högl B Metta V Sixel-Döring F Frauscher B Hulsmann J Martinez-Martin P Chaudhuri KR Parkinson’s disease sleep scale-validation of the revised version PDSS-2 Mov Disord 2011 26 644 652 21312275 10.1002/mds.23476

[B22] Martinez-Martin P Rodriguez-Blazquez C Abe K Bhattacharyya KB Bloem BR Carod-Artal FJ Prakash R Esselink RA Falup-Pecurariu C Gallardo M Mir P Naidu Y Nicoletti A Sethi K Tsuboi Y van Hilten JJ Visser M Zappia M Chaudhuri KR International study on the psychometric attributes of the non-motor symptoms scale in Parkinson disease Neurology 2009 73 1584 1591 19901251 10.1212/WNL.0b013e3181c0d416

[B23] Stern MB Marek KL Friedman J Hauser RA LeWitt PA Tarsy D Olanow CW Double-blind, randomized, controlled trial of rasagiline as monotherapy in early Parkinson’s disease patients Mov Disord 2004 19 916 923 15300656 10.1002/mds.20145

[B24] Barone P Bravi D Bermejo-Pareja F Marconi R Kulisevsky J Malagu S Weiser R Rost N Pergolide Monotherapy Study Group Pergolide monotherapy in the treatment of early PD: a randomized, controlled study Neurology 1999 53 573 579 10449123 10.1212/wnl.53.3.573

[B25] Ghys L Surmann E Whitesides J Boroojerdi B Effect of rotigotine on sleep and quality of life in Parkinson’s disease patients: post hoc analysis of RECOVER patients who were symptomatic at baseline Expert Opin Pharmacother 2011 12 1985 1998 21790503 10.1517/14656566.2011.604031

[B26] Storch A Schneider CB Wolz M Sturwald Y Nebe A Odin P Mahler A Fuchs G Jost WH Chaudhuri KR Koch R Reichmann H Ebersbach G Nonmotor fluctuations in Parkinson disease: severity and correlation with motor complications Neurology 2013 80 800 809 23365054 10.1212/WNL.0b013e318285c0ed

[B27] Lim SY Evans AH Olanow CW, Stocchi F, Lang AE Pain and paresthesia in Parkinson’s disease Parkinson’s Disease: Non-motor and Non-dopaminergic Features 2011 Oxford, UK: Blackwell Publishing Ltd 315 332

[B28] Brefel-Courbon C Payoux P Thalamas C Ory F Quelven I Chollet F Montastruc JL Rascol O Effect of levodopa on pain threshold in Parkinson’s disease: a clinical and positron emission tomography study Mov Disord 2005 20 1557 1563 16078219 10.1002/mds.20629

[B29] Hagelberg N Jääskeläinen SK Martikainen IK Mansikka H Forssell H Scheinin H Hietala J Pertovaara A Striatal dopamine D2 receptors in modulation of pain in humans: a review Eur J Pharmacol 2004 500 187 192 15464032 10.1016/j.ejphar.2004.07.024

[B30] Chaudhuri KR Martinez-Martin P Antonini A Brown RG Friedman JH Onofrj M Surmann E Ghys L Trenkwalder C Rotigotine and specific non-motor symptoms of Parkinson’s disease: post hoc analysis of RECOVER Parkinsonism Relat Disord 2013 19 660 665 23557594 10.1016/j.parkreldis.2013.02.018

