# Supplementary material for: Rotigotine transdermal system and evaluation of pain in patients with Parkinson’s disease: a post hoc analysis of the RECOVER study
Source: BMC Neurol. 2014 Mar 6;14:42. doi: 10.1186/1471-2377-14-42 (PMC4016269; doi:10.1186/1471-2377-14-42)
Supplement: Additional file 1 — Independent Ethics Committees or Institutional Review Boards of participating centres in the RECOVER study. [file 1471-2377-14-42-S1.docx]

| **Site No** | **Hospital/ Institutional Address** | **IRB Committee Chairman/ Name of Committee** |
| --- | --- | --- |
| 001 | Schloßstrasse 29  12163 Berlin  GERMANY | Local EC  Landesamt für Gesundheit und Soziales Geschäftsstelle der Ethik-  Kommission des Landes Berlin  Sächsische Strasse 28  10707 Berlin  GERMANY  Central EC  Landesärztekammer Hessen  Im Vogelsang 3  60448 Frankfurt  GERMANY |
| 002 | Parkinson Fachklinik Wolfach GmbH CoKG  Neurolog. Krankenhaus  Kreuzbergstr. 12-16  77709 Wolfach  GERMANY | Ethik-Kommission bei der Landesärztekammer Baden-Württemberg  Jahnstrasse 40  70597 Stuttgart  GERMANY  Central EC  Landesärztekammer Hessen  Im Vogelsang 3  60448 Frankfurt  GERMANY |
| 003 | Neurologische Klinik  Universität Ulm  Oberer Eselsberg 45  89081 Ulm  GERMANY | Local EC  Ethikkommission der Universität Ulm  Helmholtzstrasse 20  89081 Ulm  GERMANY  Central EC  Landesärztekammer Hessen  Im Vogelsang 3  60448 Frankfurt  GERMANY |
| 004 | Neurologische Klinik  Philipps-Universität Marburg  Rudolf-Bultmann-Str. 8  35039 Marburg  GERMANY | Local EC  Ethikkommission des Klinikums der Philipps-Universität Marburg  Baldingerstrasse 1  35033 Marburg  GERMANY  Central EC  Landesärztekammer Hessen  Im Vogelsang 3  60448 Frankfurt  GERMANY |
| 005 | Neurologisches Facharztzentrum Berlin  Am Sankt Gertrauden Krankenhaus  Paretzer Str. 12  10713 Berlin  GERMANY | Local EC  Landesamt für Gesundheit und Soziales  Geschäftsstelle der Ethik-Kommission des Landes Berlin  Sächsische Strasse 28  1070 Berlin  GERMANY  Central EC  Landesärztekammer Hessen  Im Vogelsang 3  60448 Frankfurt  GERMANY |
| 006 | Klinik u. Poliklinik f. Neurologie  Medizinische Fakultät Carl Gustav Carus  TU Dresden  Fletscherstr. 74  01307 Dresden  GERMANY | Local EC  Ethikkommission der medizinischen Fakultät  Universitätsklinik Carl Gustav Carus  Technische Universität Dresden  Fetscherstrasse 74  01307 Dresden  GERMANY  Central EC  Landesärztekammer Hessen  Im Vogelsang 3  60448 Frankfurt  GERMANY |
| 007 | Universität Leipzig  Poliklinik für Neurologie  Liebigstr. 22a  04103 Leipzig  GERMANY | Local EC  Ethik-Kommission an der Medizinischen Fakultät der Universität  Leipzig  Härtelstrasse 16 – 18  04107 Leipzig  GERMANY  Central EC  Landesärztekammer Hessen  Im Vogelsang 3  60448 Frankfurt  GERMANY |
| 008 | Paracelsus-Elena-Klinik  Klinikstrasse 16  34128 Kassel  GERMANY | Local EC  Ethik-Kommission der Landesärztekammer Hessen  Im Vogelsang 3  60448 Frankfurt  GERMANY |
| 009 | Neurologisches Krankenhaus München  Parzivalplatz 4  80804 München  GERMANY | Local EC  Ethikkommission der Fakultät für Medizin der Technischen Universität  München  Ismaninger Strasse 22  81675 München  GERMANY  Central EC  Landesärztekammer Hessen  Im Vogelsang 3  60448 Frankfurt  GERMANY |
| 011 | Dipartimento di Oncologia e Neuroscienze  Universita G. D Annunzio  Via Colle dell’Ara  66013 Chieti  ITALY | Local EC  Comitato di Etica per la Ricerca Biomedica  dell’Università degli Studi Gabriele D’Annunzio  e della ASL di Chieti  Via dei Vestini, 31  66013 Chieti  ITALY  Central EC  Comitato Etico dell’IRCCS Fondazione  Istituto Neurologico Casimiro Mondino di Pavia  Via Mondino 2  27100 Pavia  ITALY |
| 012 | Unitá Semplice Malattia di Parkinson  Clinica Neurologica C. Mondino (IRCCS)  Via Mondino, 2 (ex Via Ferrata, 6)  27100 Pavia  ITALY | Central EC  Comitato Etico dell’IRCCS Fondazione  Istituto Neurologico Casimiro Mondino di Pavia  Via Mondino 2  27100 Pavia  ITALY |
| 013 | Unitá Operativa di Neurologia  Ospedale Evangelica Valdese  Via S. Pellico, 19  10125 Torino  ITALY | Local EC  Comitato Etico Interziendale delle ASL di Torino  Piazza del Donatore di Sangue 3  10154 Torino  ITALY  Central EC  Comitato Etico dell’IRCCS Fondazione  Istituto Neurologico Casimiro Mondino di Pavia  Via Mondino 2  27100 Pavia  ITALY |
| 014 | Centro per I Disturbi del Sonno  Instituto Scientifico H S Raffaele  Via Stamira d’Ancona, 20  20127 Milano  ITALY | Local EC  Comitato Etico della ASL Città di Milano  Via Statuto 5  20121 Milano  ITALY  Central EC  Comitato Etico dell’IRCCS Fondazione  Istituto Neurologico Casimiro Mondino di Pavia  Via Mondino 2  27100 Pavia  ITALY |
| 015 | Klinika Neurologii Akademii Medycznej  Oddzial Neurologiczny  Jaczewskiego 8  20-954 Lublin  POLAND | Central EC  Komisja Bioetyczna przy  Akademii Medycznej w  Lublinie Al. Racławickie 1  20-950 Lublin  POLAND |
| 016 | Centralny Szpital Wojskowej Akademii Medycznej  Neuroogy Department  Ul. Szaserow 128  09-909 Warszawa  POLAND | Central EC  Komisja Bioetyczna przy  Akademii Medycznej w  Lublinie Al. Racławickie 1  20-950 Lublin  POLAND |
| 017 | Klinika Neurologii Doroslych  Akadamii Medycznej  Ul. Debinki 7  80-211 Gdansk  POLAND | Central EC  Komisja Bioetyczna przy  Akademii Medycznej w  Lublinie Al. Racławickie 1  20-950 Lublin  POLAND |
| 019 | Centrum Neurologii Kliniczney  Ul. Dwernickiego 8  31-530 Krakow  POLAND | Central EC  Komisja Bioetyczna przy  Akademii Medycznej w  Lublinie Al. Racławickie 1  20-950 Lublin  POLAND |
| 020 | Wojewodzki Szpital Specjalistyczny  Oddzial Neurologiczny  Zolnierska 18  10-561 Olsztyn  POLAND | Central EC  Komisja Bioetyczna przy  Akademii Medycznej w  Lublinie Al. Racławickie 1  20-950 Lublin  POLAND |
| 021 | Clinical del Rosario  Servicio de Neurologia  Principe de Vergara, 53  28006 Madrid  SPAIN | Local EC  Hospital Universitario de la Princesa  Fundación para la Investigación Biomédica (planta 1)  Secretaria del Comité Ètico de Investigación Clinica  C/Diego de León, 62  28006 Madrid  SPAIN  Central EC  Hospital Clinic i Provincial de Barcelona  Agencia de Ensayos Clinicos Escalera 8 (Sótano)  C/Villarroel, 170  08036 Barcelona  SPAIN |
| 022 | Hospital de Cruces  Servicio de Neurologia  Plaza de Cruces, s/n  48903 Barakaldo (Vizcaya)  SPAIN | Local EC  CEIC DEL HOSPITAL DE CRUCES  5a Planta del Pabellón de administración (Epidemiologia Clinica)  Plaza de Cruces, s/n  48903 Bilbao (Vizcaya)  SPAIN  Local EC  Euskadi’s Region  Secretaria Administrativa del CEIC-E  Dirección de Farmacia del Departamento de Sanidad  C/Donostia-San Sebastián, 1  01010 Vitoria  SPAIN  Central EC  Hospital Clinic i Provincial de Barcelona  Agencia de Ensayos Clinicos Escalera 8 (Sótano)  C/Villarroel, 170  08036 Barcelona  SPAIN |
| 023 | Hospital Ramón y Cajal  Servicio de Neurologia  Ctra. De Colmenar Viejo Km 9,1  28034 Madrid  SPAIN | Local EC  Comité Ètico de Investigación Clinica  Hospital Ramón y Cajal  Ctra. Colmenar Km 9, 100  28034 Madrid  SPAIN  Central EC  Hospital Clinic i Provincial de Barcelona  Agencia de Ensayos Clinicos Escalera 8 (Sótano)  C/Villarroel, 170  08036 Barcelona  SPAIN |
| 024 | Hospital de la Ribera  Unidad del Sueño  Carretera de Corbera Km 1, Sotano  46600 Alzira (Valencia)  SPAIN | Local EC  Comité Ètico de Investigación Clinica  Hospital de La Ribera  Laboratorio 2a Planta  Ctra. Corbera Km. 1  46600 Alzira (Valencia)  SPAIN  Central EC  Hospital Clinic i Provincial de Barcelona  Agencia de Ensayos Clinicos Escalera 8 (Sótano)  C/Villarroel, 170  08036 Barcelona  SPAIN |
| 025 | Hospital Clinic I Provincial de Barcelona  Servicio de Neurologia (Escalera 8, planta 4)  C/Villarroel 170  08036 Barcelona  SPAIN | Central EC  Hospital Clinic i Provincial de Barcelona  Agencia de Ensayos Clinicos Escalera 8 (Sótano)  C/Villarroel, 170  08036 Barcelona  SPAIN |
| 027 | Novant Medical Group, Inc.  (d.b.a.) Central Carolina Neurology & Sleep  911 W. Henderson Street  Suite L30  28144 Salisbury, NC  USA | Central EC  Copernicus Group IRB  One Triangle Drive Suite 100  P.O. Box 110605  Research Triangle Park  NC 27709  USA |
| 028 | The Institute of Sleep Medicine, Inc.  7500 San Felipe  Suite 525  77063 Houston, TX  USA | Central EC  Copernicus Group IRB  One Triangle Drive Suite 100  P.O. Box 110605  Research Triangle Park  NC 27709  USA |
| 029 | Suncoast Neuroscience Associates, Inc.  2201 Central Ave, Suite 301  33713 St. Petersburg, FL  USA | Central EC  Copernicus Group IRB  One Triangle Drive Suite 100  P.O. Box 110605  Research Triangle Park  NC 27709  USA |
| 031 | Concord Repatriation General Hospital  Neurosciences Unit, Department of Neurology  Level 5 West  Hospital Road  Concord 2139 NSW  AUSTRALIA | Local EC  Human Research Ethics Committee  Concord Repatriation General Hospital (CRGH)  Concord Research Office  Level 1, Building 75  Hospital Road  Concord NSW 2139  AUSTRALIA |
| 032 | Watkins Medical Centre  Level 10  225 Wickham Terrace  4000 Brisbane QLD  AUSTRALIA | Local EC  Uniting Healthcare Human Research Ethics Committee  1st Floor Moolands House  The Wesley Hospital  451 Coronation Street  Auchenflower QLD 4066  AUSTRALIA |
| 033 | Royal Adelaide Hospital  Department of Neurology  Level 5, Emergency Block  North Terrace  Adelaide 5000 SA  AUSTRALIA | Local EC  Royal Adelaide Hospital  Research Ethics Committee  Level 3, Hanson Institute  North Terrace  Adelaide SA 5000  AUSTRALIA |
| 034 | St. Vincent’s Hospital  Department of Clinical Neurosciences and Neurological Research  Level 5, Daly Wing  35 Victoria Parade  Fitzroy Victoria 3065  AUSTRALIA | Local EC  Human Research Ethics Committee – D  St. Vincent’s Hospital (Melbourne) Limited  Research and Grants Unit  41 Victoria Parade  Fitzroy Vic 065  AUSTRALIA |
| 035 | Samodzielny Publiczny Szpital Kliniczny  Oddzial Kliniczny Neurologii i Epileptologii  Ul. Czerniakowska 231  00-416 Warszawa  POLAND | Central EC  Komisja Bioetyczna przy  Akademii Medycznej w  Lublinie Al. Racławickie 1  20-950 Lublin  POLAND |
| 036 | Universitätsklinikum  Universitätsklinik für Neurologie  Anichstr. 25  6020 Innsbruck  AUSTRIA | Central EC  Ethikkommission der Medizinischen Universität Innsbruck  Innrain 63  6020 Inssbruck  AUSTRIA |
| 037 | Katedra I Klinika Neurologii PAM  Ul. Unii Lubelskiej 1  71-252 Szczecin  POLAND | Central EC  Komisja Bioetyczna przy  Akademii Medycznej w  Lublinie Al. Racławickie 1  20-950 Lublin  POLAND |
| 038 | Hyvinkään sairaala  Neurolgian poliklinikka  Sairaalankatu 1  05850 Hyvinkää  FINLAND | Central EC  Pohjois-Pohjanmaan  Sairaanhoitopiirin eettinen toimikunta  Hallintokeskus  Kajaanintie 50  90220 Oulu  FINLAND |
| 039 | Oulu University Hospital  Neurolgian poliklinikka  Kajaanintie 50  90220 Oulu  FINLAND | Central EC  Pohjois-Pohjanmaan  Sairaanhoitopiirin eettinen toimikunta  Hallintokeskus  Kajaanintie 50  90220 Oulu  FINLAND |
| 042 | Colorado Neurological Institute  701 East Hampden Avenue  Suite 510  2424 Englewood, CO 80113  USA | Local EC  HCA –Health One IRB  720 South Colorado Blvd.  Suite 230A  Glendale  CO 80246  USA |
| 043 | Parkinson’s Disease & Movement Disorders Center  NeuroHealth  227 Centerville Road  02886 Warwick, RI  USA | Central EC  Copernicus Group IRB  One Triangle Drive Suite 100  P.O. Box 110605  Research Triangle Park  NC 27709  USA |
| 044 | Jakobstrasse 26  06618 Naumburg  GERMANY | Local EC  Ethik-Kommission des Landes Sachsen-anhalt  Kühnauerstrasse 70  06846 Dessau  GERMANY  Central EC  Landesärztekammer Hesser  Im Vogelsang 3  60448 Frankfurt  GERMANY |
| 045 | Granseerstrasse 23b  16515 Oranienburg  GERMANY | Local EC  Landesärztekammer Brandenburg  Ehtik-Kommission  Dreiferstrasse 12  03044 Cottbus  GERMANY  Central EC  Landesärztekammer Hesser  Im Vogelsang 3  60448 Frankfurt  GERMANY |
| 046 | Hazel Hutchman Neurosearch, Inc.  19231 Victory Blvd.  Suite 355 North Wing  91335 Reseda, CA  USA | Central EC  Copernicus Group IRB  One Triangle Drive Suite 100  P.O. Box 110605  Research Triangle Park  NC 27709  USA |
| 047 | Debreceni Egyetem Orvos – és Egészségtudományi Centrum  Neurológiai Klinika  Nagyerdei krt. 98  4012 Debrecen  HUNGARY | Central EC  ETT-KFEB  Arany Janos u. 6-8  1051 Budapest  HUNGARY |
| 048 | Szabolcs-Szatmár-Bereg Megyei Önkormányzat Jósa András Kórház  Neurologiai Osztaly  Szent Istvan u. 68  4400 Nyiregyhaza  HUNGARY | Central EC  ETT-KFEB  Arany Janos u. 6-8  1051 Budapest  HUNGARY |
| 049 | Zala Megyei Kórház  Neurológiai Osztály  Zrinyi M u. 1  8900 Zalaegerszeg  HUNGARY | Central EC  ETT-KFEB  Arany Janos u. 6-8  1051 Budapest  HUNGARY |
| 050 | Fővárosi Önkormányzat Uzsoki utcai Kórház  Neurológiai Osztály  Uzsoki u. 29-45  1145 Budapest  HUNGARY | Central EC  ETT-KFEB  Arany Janos u. 6-8  1051 Budapest  HUNGARY |
| 053 | Van der Veer Institute for Parkinsons & Brain Research  66 Stewart Street  Christchurch  NEW ZEALAND | Central EC  Multi Region Ethics Committee  Ministry of Health  PO Box 5013  Wellington  NEW ZEALAND |
| 054 | Wellington Hospital  Department of Clinical Neurosciences and Neurological Research  Riddiford Street, Newtown  Wellington  NEW ZEALAND | Central EC  Multi Region Ethics Committee  Ministry of Health  PO Box 5013  Wellington  NEW ZEALAND |
| 055 | Tygerberg Hospital, Dept. of Neurology  Ward A8  Francie van Zyl Road  7050 Cape Town  SOUTH AFRICA | Local EC  University of Stellenbosch Committee  for Clinical Trials (CCT)  Francie van Zijl Drive  7505 Cape Town  SOUTH AFRICA |
| 056 | Constantiaberg Medi-Clinic  Plumstead  106 Bumham Rd  7800 Cape Town  SOUTH AFRICA | Local EC  South African Medical Association  Research Ethics Committee (SAMAREC)  Block F, Castle Walk Corporate Park  Nossob Street  Erasmuskloof Ext 3  Pretoria 0153  SOUTH AFRICA |
| 057 | Groote Schuur Hospital  E8-75 Neurology Unit  Observatory  7925 Cape Town  SOUTH AFRICA | Local EC  University of Cape Town  Health Science Faculty  Research Ethics Committee  Room E-24-54 Groote Schuur Hospital, Old Main Building  Observatory 7925  Cape Town  SOUTH AFRICA |
| 058 | Wilgers Medical Centre  538 Denneboom Road  0041 Pretoria / Gauteng  SOUTH AFRICA | Local EC  South African Medical Association  Research Ethics Committee (SAMAREC)  Block F, Castle Walk Corporate Park  Nossob Street  Erasmuskloof Ext 3  Pretoria 0153  SOUTH AFRICA |
| 059 | Rosebank  29 Jellicoe-Avenue  2196 Johannesburg  SOUTH AFRICA | Local EC  South African Medical Association  Research Ethics Committee (SAMAREC)  Block F, Castle Walk Corporate Park  Nossob Street  Erasmuskloof Ext 3  Pretoria 0153  SOUTH AFRICA |
| 060 |  | Central EC  Copernicus Group IRB  One Triangle Drive Suite 100  P.O. Box 110605  Research Triangle Park  NC 27709  USA |
| 061 | Bupa Fylde Coast Hospital  St. Walburgas Road  Lancashire  Blackpool  FY3 8BP  UNITED KINGDOM | Local EC  Cumbria and Lancashire Research Ethics Committee A  North West Centre of Research Ethics Committees  Room 181, 1st Floor  Gateway House  Piccadilly South  Manchester, M60 7LP  UNITED KINGDOM  Central EC  South East Research Ethics Committee  South East Coast Strategic Health Authority  Preston Hall  Aylesford  Kent, ME20 7NJ  UNITED KINGDOM |
| 062 | University Hospital Lewisham  Lewisham High Street  London  SE13 6LH  UNITED KINGDOM | Local EC  Lewisham Research Ethics Committee  South London REC Office – 1st Floor  Owen Centre  University Hospital Lewisham  Lewisham High Street  London, SE13 6LH  UNITED KINGDOM  Central EC  South East Research Ethics Committee  South East Coast Strategic Health Authority  Preston Hall  Aylesford  Kent, ME20 7NJ  UNITED KINGDOM |
| 063 | The Walton centre of Neurology, Neurosurgery  Lower Lane Fazakerley  L9 7LJ Liverpool  UNITED KINGDOM | Local EC  Sefton Research Ethics Committee  Research Ethics Office  Victoria House  Bishop Goss Complex  Rose Place  Liverpool, L3 3AN  UNITED KINGDOM  Central EC  South East Research Ethics Committee  South East Coast Strategic Health Authority  Preston Hall  Aylesford  Kent, ME20 7NJ  UNITED KINGDOM |
| 064 | North Tyneside General Hospital  Rake Lane  North Shields  Tyne & Wear  NE29 8NH  UNITED KINGDOM | Local EC  Newcastle & North Tyneside LREC  Room G14 Dental School  Framlington Place  Newcastle, NE2 4HH  UNITED KINGDOM  Central EC  South East Research Ethics Committee  South East Coast Strategic Health Authority  Preston Hall  Aylesford  Kent, ME20 7NJ  UNITED KINGDOM |
| 066 |  | Local EC  Frenchay LREC  Pembroke Room  Beaufort House  Southmead Hospital  Westbury-on-Trym  Bristol, BS10 5NB  UNITED KINGDOM  Central EC  South East Research Ethics Committee  South East Coast Strategic Health Authority  Preston Hall  Aylesford  Kent, ME20 7NJ  UNITED KINGDOM |
| 067 | Hajdú-Bihar Megyei  Önkormányzat Kenézy Gyula Kórháza  Ideggyógyászat  Bartók Béla út. 2-26  4043 Debrecen  HUNGARY | Central EC  ETT-KFEB  Arany Janos u. 6-8  1051 Budapest  HUNGARY |
| 068 | Pécsi Tudományegytem  Neurológiai Klinika  Rét u. 2  7623 Pécs  HUNGARY | Central EC  ETT-KFEB  Arany Janos u. 6-8  1051 Budapest  HUNGARY |
| 069 | Wake Forest University Health Sciences  Department of Neurology  Medical Center Boulevard  Winston-Salem, NC 27157  USA | Local EC  Wake Forest University Health Services IR  117 E. Reynolds Hall  PO Box 7528  Winston-Salem  NC 27109  USA |
| 070 | Neurosearch II, Inc.  2781 Loma Vista Road  Suite C  Ventura, CA 93003  USA | Central EC  Copernicus Group IRB  One Triangle Drive Suite 100  P.O. Box 110605  Research Triangle Park  NC 27709  USA |
