# Supplementary material for: Rotigotine transdermal system and evaluation of pain in patients with Parkinson’s disease: a post hoc analysis of the RECOVER study
Source: BMC Neurol. 2014 Mar 6;14:42. doi: 10.1186/1471-2377-14-42 (PMC4016269; doi:10.1186/1471-2377-14-42)
Supplement: Additional file 2: Table S1 — Pearson correlation coefficients (r) for change from baseline in Likert pain scale score with UPDRS III and PDSS-2 change from baseline in rotigotine-treated patients. [file 1471-2377-14-42-S2.doc]

**Additional file 2: Table S1.** **Pearson correlation coefficients (*r*) for change from baseline in Likert pain scale score with UPDRS III and PDSS-2 change from baseline in rotigotine-treated patients**

|  | **‘Any’ pain (n = 187)**  (pain score ≥1) | |
| --- | --- | --- |
|  | **‘Mild’ pain (n = 87)** (pain score 1–3) | **‘Moderate-to-severe’ pain (n = 100)** (pain score ≥4) |
| Likert pain scale vs.  UPDRS III total score | *r* = 0.33 (p = 0.0001) | |
|  | *r* = 0.40 (p = 0.001) | *r* = 0.31 (p = 0.011) |
| Likert pain scale vs.  PDSS-2 total score | *r* = 0.17 (p = 0.045) | |
|  | *r* = 0.30 (p = 0.018) | *r* = 0.16 (p = 0.191) |

PDSS-2: Parkinson’s Disease Sleep Scale; UPDRS III: Unified Parkinson’s Disease Rating Scale.
